# The impact of PM_2.5_ on mortality in older adults: evidence from retirement of coal-fired power plants in the United States

**DOI:** 10.1186/s12940-020-00573-2

**Published:** 2020-03-03

**Authors:** Maoyong Fan, Yi Wang

**Affiliations:** 1 Department of Economics, Miller College of Business, Ball State University, 2000 W. University Ave. WB 201, Muncie, IN 47306 USA; 2 Department of Environmental Health, Fairbanks School of Public Health, Indiana University, Indianapolis, Indiana USA

**Keywords:** PM_2.5_, Coal-fired Power Plants, Natural Experiment, Causal Relationship, Mortality, Older Adults

## Abstract

**Background:**

Evidence of causal relationship between mortality of older adults and low- concentration PM_2.5_ remains limited.

**Objectives:**

This study investigates the effects of low-concentration PM_2.5_ on the mortality of adults older than 65 using the closure of coal-fired power plants in the Eastern United States as a natural experiment.

**Methods:**

We investigated power plants in the Eastern United States (US) that had production changes through unit shutdown or plant retirement between 1999 and 2013. We included only non-clustered power plants without scrubbers and with capacities greater than 50 MW. We used instrumental variable (IV) and difference-in-differences (DID) approaches to estimate the causal impact of PM_2.5_ concentrations on mortality among Medicare beneficiaries. We compared changes in monthly age-adjusted mortality before and after the retirement of coal-fired plants between the treated and control counties; we accounted for annual wind direction in our selection of treated and control counties. In the models, we initially included only county and monthly fixed effects, and then adjusted for covariates including: 1) only weather variables (temperature, dew point, pressure); and 2) weather variables and socio-economic variables (median household income and poverty rate).

**Results:**

The monthly age-adjusted mortality rate averaged across all plants was approximately 423 per 100,000 (SD = 69) and was higher for males than females. Mean PM_2.5_ concentrations across all counties were 12 μg/m^3^ (SD = 3.78). Using the IV method, we found that reductions in PM_2.5_ concentrations significantly decreased monthly mortality among older adults. IV results show that a 1-μg/m^3^ reduction in PM_2.5_ concentrations leads to 7.17 fewer deaths per 100,000 per month, or a 1.7% lower monthly mortality rate among people older than 65 years. Using the DID approach, we found that power plant retirement significantly decreased: 1) monthly PM_2.5_ levels by 2.1 μg/m^3^, and 2) monthly age-adjusted mortality by approximately 15 people per 100,000 (or 3.6%) in treated counties relative to control counties. The mortality effects were higher among males than females and its impact was the greatest among people older than 75 years.

**Conclusion:**

These findings provide evidence of the effectiveness of local, plant-level control measures in reducing near-plant PM_2.5_ and mortality among U.S. Medicare beneficiaries.

## Background

A large number of observational epidemiological studies have established a positive association between short-term (e.g. daily or monthly) exposure to fine particulate matter (PM_2.5_) and mortality in the past [1–7]. Recent studies have shown that mortality was positively linked to PM_2.5_ concentrations that were lower than the current EPA standard (12 μg/m^3^) [8, 9]. However, the observed association between PM_2.5_ and mortality may result from factors other than air pollution. For example, sharp changes in air pollution levels are often driven by local weather conditions rather than changes in polluting activities. If weather conditions cause health problems through other channels, researchers have to separate out the effects of elevated air pollution on health from the weather effects. Lacking appropriate control groups makes it difficult to rule out alternative explanations in most observational studies. Longitudinal studies can also suffer from selection bias: study participants self-select into different locations; thus, their exposure to different levels of pollution is endogenous. Wealthy people, whose health status tends to be good for other reasons, can migrate to clean regions, while poor people may be confined to polluted areas. Consequently, these observational/associational approaches tend to produce unreliable estimates due to endogeneity issues [10]. Accurately estimating the health effects of PM_2.5_ is critical for assessing optimal environmental policies. Overstating the impacts will lead to over-stringent environmental regulation and potentially hurt economic growth, while underestimating the impacts will provide less than optimal protection for vulnerable people (e.g. infants and the elderly) and result in significant welfare losses.

Coal power plants are major contributors to ambient air pollution. A typical uncontrolled (without scrubbers) coal plant (Typical plant assumptions: Capacity = 600 MW; Capacity Factor = 69%; Heat Rate = 10,415; CO2 Emissions Rate = 206 pounds of CO2/Million Btu.) emits 500 tons of small airborne particles each year. Since 2009, coal plant retirement plans have been announced across the United States due primarily to increased environmental regulations and lower prices of natural gas: these resulted in a large number of coal power plants retired from the end of 2007 through 2012. A complete list of coal plant retirements is available at: http://www.sourcewatch.org/index.php/Coal_plant_retirements.

We use the retirement of coal plants as a natural experiment to estimate the causal effects of PM_2.5_ on mortality among Medicare beneficiaries. We identified five large power plants which retired during the sample period in the Eastern United States. The retirement of these plants resulted in a sudden improvement in air quality in downwind counties. The relatively rapid reduction of major pollution sources yielded a relatively rapid reduction in mortality attributed to coal power plants; this provided a favorable setting for us to conduct a natural experiment evaluating the causal effects of PM_2.5_ on mortality. By comparing the mortality rates in the affected counties with similar but unaffected counties before and after the plant retirement, we are able to estimate the effect of air pollution on the mortality rates among Medicare beneficiaries.

Our study contributes to the literature by evaluating the causal effect of low PM_2.5_ concentrations on mortality among older adults in the United States. To estimate the health effects of low PM_2.5_ concentrations, we combined the retirements of five large coal plants with death records data from the Center for Medicare and Medicaid Services (CMS). To the best of our knowledge, this is the first study that examines the impact of coal plant retirements on mortality rates among the elderly. We conducted a variety of robustness checks and find our conclusions are unaltered. The evidence suggests that our research design provides a credible basis for evaluating the causal effect of PM_2.5_ on health.

## Research design

We collected information on all coal-fired power plants in the Eastern US and performed a thorough investigation of power plant retirements between 1999 and 2013. We selected power plants that had experienced unit shutdown or plant retirement, marked by an exact date of the change (rather than an estimated period leading up to the change). In our analysis, only production changes in or after 2008 and before December 2013 are included because that is the period when a large number of coal plants were retired, mainly due to two main converging factors. The first are new and proposed US Environmental Protection Agency (EPA) regulations, including: a) the proposed Clean Air Transport Rule, b) the proposed Coal Combustion Residuals rule, c) the proposed Tailoring Rule (covering greenhouse gas emissions), d) the Ozone National Ambient Air Quality Standards (NAAQS), e) the forthcoming National Emission Standard for Hazardous Air Pollutants (NESHAPs), and f) cooling water regulations under section 316(b) of the Clean Water Act. The other factor was the decreased price of natural gas that made operating old coal plants much less economical. This selection process resulted in five large retired power plants in the Eastern US for our empirical analysis.

### Data sources

We acquired daily mortality along with gender and age group data of U.S. residents who were 65 and older between 1999 and 2013 for all counties from the enrollment file from CMS. We obtained annual county population data from the U.S. Census Bureau and calculated the weights of different age groups. We used CMS’ SSA to FIPS State and County Crosswalk to create a linkage between mortality data and population data, and calculated the age-adjusted mortality rate per 100,000 in each county.

The power plants were selected from a list of coal-fired power plants from Clean Air Watch (https://www.sourcewatch.org/index.php/Clean_Air_Watch), which monitors all U.S. coal plants and their activities. We identified the exact time (month and year) of unit shutdown or plant retirement, and verified it using data cross-referenced from the Federal Register, the EPA website and reports, Clean Air Watch, and local policy and neighborhood reports. Plants whose dates could not be verified were not included in our analysis. Data on production capacity, geographical coordinates, and scrubber installation are also retrieved from the same site. We obtained ambient PM_2.5_ monitoring data from the EPA Air Quality System, weather variables from the National Climatic Data Center at the National Oceanic and Atmospheric Administration (NOAA), and wind direction from the WindFinder website using a wind rose (www.windfinder.com).

We collected county-level socioeconomic variables from the US Bureau of Labor Statistics, the U.S. Census Bureau, and the American Community Survey, including median household income, poverty rate, percentage of non-Hispanic Whites in the population, and percentage of population with college degree. After eliminating counties with missing mortality data, PM_2.5_ values, weather data, and socioeconomic variables, we ended up with 770 and 7504 county-month observations for the treated and control groups, respectively.

### Estimation strategy

Our empirical strategy involves three steps. First, we identified the treatment counties based on the location of the power plant and wind direction. We only included large power plants that were: 1) not clustered (plants which have overlapping treatment counties within their respective 50 km radius are considered a cluster), and 2) had production capacities greater than 50 MW (MW). The size restriction was because of the greater impact that large plants have upon air quality than those with smaller capacities. We excluded plants that had installed scrubbers. For each coal-fired plant, we identified the location and the nearest weather station, from which we obtained annual prevailing wind direction. For each county we identified the coordinates of the county seat – the capital city of a county where in most cases the majority of the county population resides – and calculated the distance between the power plants and county seats. Additionally, we identified counties downwind of the power plants as potential candidates for treated counties. We checked the distance from the power plant to potential candidates and only included counties within a 50 km radius of the candidate plants as our treatment counties. We identified and included five plants in the final analysis, listed in Table 1. We also include a map showing the locations of the plants in Appendix Fig. 3.

**Table 1:** Power Plants Included in the Analysis

State	County	Plant	Capacity (MW)	Exact Retirement Time
Georgia	Putnam	Harllee Branch Generating Plant	359	September, 2013
North Carolina	Rockingham	Dan River Steam Station	70	April, 2012
Ohio	Montgomery	Hutchings Station	69	June, 2013
Tennessee	Hawkins	John Sevier Fossil Plant	200	December, 2012
Tennessee	Rhea	Watts Bar Fossil Plant	60	December, 2011

Second, for each treated county, we used covariate matching (CVM) based on county characteristics to select controls from over 2000 US counties. The basic idea of matching is to mimic an experimental setting by finding untreated counties with similar characteristics to the treated counties [11–13]. Intuitively, comparing two counties with the same predetermined characteristics, where one is treated and the other is not, is like comparing these two counties in a randomized experiment. However, not all non-treated counties can be used as a potential control. To avoid potential contamination, we excluded all counties located within a 50 km radius of any coal power plant from the potential control group. During the matching process, each treated county was matched to several closest counties in the control group. The matching is based on the distance measured by the vector norm ‖∙‖. Let ‖*x*‖_*V*_ = (*x*^′^*Vx*)^1/2^ be the vector norm with the positive definite matrix *V* (we used the diagonal matrix, of which the diagonal elements are the inverses of the variances of *Xi* (the element of the set of covariates), as our weighting matrix V. The weighting matrix *V* accounts for the difference in the scale of the covariates). The CVM defines ‖*z* − *x*‖_*V*_ as the distance between the vector *x* and *z*, where *x* and *z* represent the covariates for treated counties and a potential match. The matching variables we used were median household income, poverty rate, percentage of non-Hispanic White population, percentage of the population with college degrees, and census region. For each treated county, we selected 10 control counties that were most similar based on the values of these indicators averaged over the sample period.

Finally, after constructing a set of treatment and control counties, we estimated the effects of air pollution on monthly age-adjusted mortality using the instrumental variable (IV) approach. We compared changes in monthly age-adjusted mortality before and after the retirement of coal plants between the treated and control counties. Initially, we controlled only for county and month fixed effects. In subsequent estimations we adjusted for other covariates including: a) weather variables (temperature, dew point, and pressure); and b) time-varying socioeconomic variables mentioned above (i.e., median household income and poverty rate). The county fixed effects absorb time-constant confounding factors. Besides the IV approach, we also used the differences-in-differences (DID) approach to estimate the impact of coal plant retirements on PM_2.5_ and mortality among populations older than 65.

## Econometric models

First, we used IV models to estimate the impact of PM_2.5_ on mortality. Our analysis compares changes in mortality rates in counties that experienced reductions in PM_2.5_ with those in counties that experienced little or no reduction in PM_2.5_ concentrations. We estimated the effects of PM_2.5_ on mortality rates using a two-way fixed-effects IV model:

1 $$ {P}_{it}=\gamma {R}_{it}+{X}_{it}^{\prime}\theta +{\tau}_i+{\pi}_t+{\xi}_{it} $$

2 $$ {Y}_{it}=\delta {\hat{P}}_{it}+{X}_{it}^{\prime}\beta +{u}_i+{v}_t+{\varepsilon}_{it} $$

where *P*_*it*_ is the PM_2.5_ level in county *i* at time *t* and *Y*_*it*_ is the monthly age-adjusted mortality rate per 100,000 in county *i* at time *t*. *X*_*it*_ is a vector of weather conditions and socioeconomic factors, which are also likely to affect the mortality rate. *u*_*i*_ and *τ*_*i*_ are county fixed effects included to capture county-specific and time-invariant differences in mortality rate, *v*_*t*_ and *π*_*t*_ are year-month fixed effects to capture common health shocks, and *ε*_*it*_ and *ξ*_*it*_ are unobservable disturbances. *R*_*it*_ is a post-retirement indicator that equals 1 for treated county *i* in the post-retirement period and is 0 otherwise. *R*_*it*_ is the IV that causes changes in PM_2.5_ without directly affecting mortality. Standard errors are clustered at the state level to allow arbitrary correlation over time within a state.

In the first stage, we estimated Eq. (1) to estimate the effects of power plant retirement upon PM_2.5_. The coefficient *γ* is essentially a difference-in-difference estimator, capturing differences in changes of PM_2.5_ levels after the power plant retirement between the treatment and control counties. We expect *γ* to be negative. In the second stage of the IV regression (Eq. (2)), we estimated the effect of air pollution on mortality. If PM_2.5_ negatively affects health, we expect that fewer people will die in treated counties than in control counties after retirement of power plants.

We also employed the difference-in-differences approach to estimate the causal impact of coal-fired power plant retirement on PM_2.5_ and mortality directly [14, 15]. We compared changes in PM_2.5_ and monthly age-adjusted mortality rates before and after the retirement of coal-fired plants between the treated and control counties. Specifically, we estimated the following DID model:

3 $$ {O}_{it}=\rho T{R}_{it}+{X}_{it}^{\hbox{'}}\beta +{c}_i+{m}_t+{\mu}_{it} $$

where *O*_*it*_ is either air pollution level or mortality rate. *TR*_*it*_ is the interaction term of the treatment indicator and the post-retirement indicator. *X*_*it*_ is defined the same as Eqs. (1) and (2). The parameter *ρ* represents the impact of power plant retirement on PM_2.5_ or mortality caused by the retirement of coal-fired boilers in power plants. *c*_*i*_ is the county fixed effect, *m*_*t*_ is the year-month fixed effect, and *μ*_*it*_ is the error term. Standard errors are clustered at the state level.

### Heterogeneous effects

We examined the following heterogeneous effects. First, to account for the gender difference, we analyzed males and females separately. Second, we investigated the nonlinear effects across age groups by examining the impact of air pollution on mortality rates separately for people aged 65–75 and people older than 75.

### Robustness checks

We conducted various robustness checks. First, if changes in smoking rates coincide with the retirement of the coal-fired power plants, omitting the variable will cause bias. To make sure that smoking rates do not affect our results, we add county-level annual smoking rates as a control variable. Second, to make sure that each treated county is compared with the most similar control counties, we reduced the number of matched control counties for each treated county. Third, to increase the size of the treated group, we included in the sample those power plants that had scrubbers. Finally, to reduce the efficiency loss, we excluded those counties with less than 60 months of data.

## Results

Table 2 summarizes the descriptive statistics of mortality rates, PM_2.5_ concentrations, weather conditions, and socioeconomic variables across all counties. The age-adjusted monthly mortality rate averaged across all counties is approximately 423 per 100,000 with a standard deviation of 69. The male mortality rate is higher than the female rate. Age-adjusted mortality rates in treated counties are slightly higher than those in control counties and in the full sample. The mean PM_2.5_ across all counties is 12.04 μg/m^3^ (SD = 3.78 μg/m^3^) and the treated county mean PM_2.5_ is roughly 2 μg/m^3^ higher with similar variability. The statistics for dew point and pressure are similar across treated and control counties and the full sample. Treated and control counties have similar median household income and poverty rate.

**Table 2:** Summary Statistics of the Main Variables

	Full Sample		Treated Counties		Control Counties	
	Mean	S.D.	Mean	S.D.	Mean	S.D.
Age-Adjusted Mortality Rate	423.0	68.99	444.4	68.13	420.8	68.70
Male Age-Adjusted Mortality Rate	458.2	97.15	485.7	96.00	455.4	96.83
Female Age-Adjusted Mortality Rate	400.4	75.74	419.4	72.24	398.4	75.83
Mortality Rate 65–75	193.9	54.82	206.4	54.53	192.6	54.69
Mortality Rate 75+	694.3	118.5	726.5	114.2	691.0	118.4
PM_2.5_ (μg/m^3^)	12.04	3.782	14.02	4.059	11.84	3.693
Temperature (°F)	59.46	16.89	59.53	15.08	59.45	17.07
Dew Point (°F)	47.86	16.32	47.77	14.78	47.87	16.47
Barometric Pressure (Hg)	29.31	0.790	29.20	0.417	29.32	0.818
Median Household Income ($1000)	41.60	7.045	40.42	4.705	41.72	7.232
Poverty Rate (%)	15.61	4.087	15.81	4.466	15.59	4.046
Observations	8274		770		7504	

Notes: All variables are measured at the county monthly level. We use the age structure in the 2010 Census to calculate the age-adjusted mortality rates. Mortality rate is reported as deaths per 100,000 people

**Table 3:** The Effect of PM2.5 on Monthly Mortality Rate

	First Stage			Second Stage		
	PM2.5 (μg/m3)			Mortality Rate		
	(1)	(2)	(3)	(4)	(5)	(6)
Shut Down Indicator	−2.11*** (− 2.78 - -1.44)	−2.13*** (− 2.82 - -1.44)	−2.13*** (− 2.84 - -1.42)			
PM_2.5_ (μg/m^3^)				6.62*** (3.05–10.19)	6.45*** (2.93–9.96)	7.17*** (3.50–10.85)
Weather Controls	N	Y	Y	N	Y	Y
Socioeconomic Controls	N	N	Y	N	N	Y
County Fixed Effects	Y	Y	Y	Y	Y	Y
Month Fixed Effects	Y	Y	Y	Y	Y	Y
F-Statistics	36.43	41.99	36.93	–	–	–
Observations	8274	8274	8274	8274	8274	8274

Notes: This table reports the instrumental variable regression coefficients and standard errors. Retirement of coal-fired power plants is used as the instrumental variables for monthly PM_2.5_ concentrations. The dependent variable is the monthly standardized mortality rate per 100, 000 people. Columns 1–3 report the first stage regression results, and columns 4–6 report the second stage results. Weather controls include temperature, dew point, and barometric pressure. Socioeconomic controls include median household income and poverty rate. Standard errors are clustered at the state level. * *p* < 0.10, ** *p* < 0.05, *** *p* < 0.01

### Graphic analysis

**Fig. 1:**
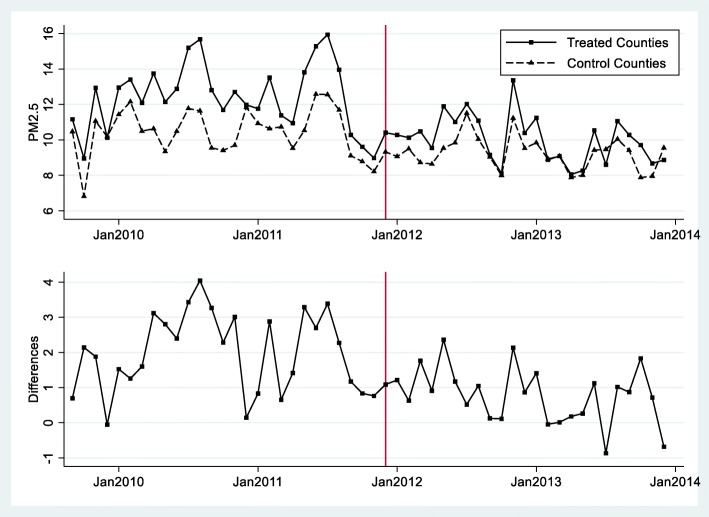
Changes of PM_2.5_ before and after earliest month of retirement between treatment and control counties Notes: The figure depicts the time trend of monthly PM_2.5_ concentrations for the treatment and control counties separately. In the upper graph, the solid line represents the treated counties and the dashed line represents the control counties. The lower graph plots the differences. December 2011 is the month when Watts Bar Fossil Plant in Tennessee shut down. The other four coal power plants shut down later (see Table 1). This figure shows that air quality in the treated counties after the retirement of a nearby power plant was improved, while the air pollution levels in the control cities were similar over time.

**Fig. 2:**
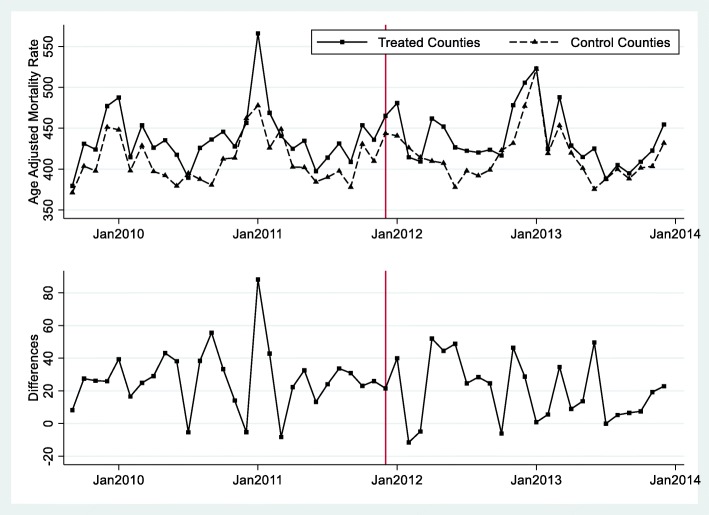
Changes of monthly age-adjusted mortality before and after earliest month of retirement between treatment and control counties Notes: The figure shows the differences in the age-adjusted mortality rates between treated and control counties over time. In the upper graph, the solid line represents the treated counties and the dashed line represents the control counties. The lower graph plots the differences. December 2011 is the month when Watts Bar Fossil Plant in Tennessee shut down. The other four coal power plants shut down later (see Table 1). This figure shows that the age-adjusted mortality rate in the treated counties after the retirement of a nearby power plant was lower, while the mortality rate in the control cities was similar over time.

Figure 1 and Fig. 2 depict the differences in PM_2.5_ and mortality rates, respectively, between the treated and control counties during 2009–2013. In the top plot of each figure, the solid line represents the trend for treated counties and the dashed line represents the trend for control counties. The bottom plot of each figure shows the differences (treated minus control) between the two groups. The vertical line indicates the earliest month (December 2011) when coal power plant retirement occurred among all five power plants. In Fig. 1, we observed a strong seasonal pattern in the trends of air quality for both the treated and control groups. In the control group, air quality was relatively stable from year to year. The average PM_2.5_ concentrations were higher in the treated group than in the control group before the power plant retirement. In contrast, we observe a lower level of PM_2.5_ concentrations after the power plant retirement for the treated group. In Fig. 2, we also observed a trend of stable differences in mortality rate between treated and control counties before the earliest retirement month and reduced differences after that. This is consistent with our natural experimental design: after a power plant retired, the treated counties experienced a reduction in PM_2.5_ and mortality.

### Validity of the instrumental variable

The first requirement of a valid instrument is its power to predict endogenous variable. Columns (1)–(3) of Table 2 report the results from estimating Eq. (1). We found that the coefficients of power plant retirement is statistically significant and the F-statistics exceed the conventional criterion (i.e., 10) for a powerful first stage in all models [16].

The second requirement for a valid IV is that the IV should affect the outcome only through its impact on the endogenous variable, i.e., the IV is excludable from the second-stage regression [15]. While this requirement cannot be credibly tested, we provide some evidence suggesting that the IV is exogenous. We found that the estimated coefficients of the IV in Table 2 are remarkably stable across different specifications. Presumably, the weather and socioeconomic variables are important determinants for mortality. However, including these controls has little impact on the first-stage regression results. The results suggest that the IV is orthogonal to weather and local socioeconomic conditions.

### Empirical analysis

Table 2 presents the results from estimating Eqs. (1) and (2) using two-stage least square regression. The first three columns are estimates from the first stage. In column (1), we estimated the effect of the power plant retirement on PM_2.5_ concentrations using Eq. (1). Column (1) includes no control variables. Columns (2) and (3) gradually add weather and socioeconomic controls. We included temperature, dew point and barometric pressure as weather controls. Air pollution tended to increase on both extremely hot and cold days, due to excessive energy consumption. At the same time, people were more likely to die on both extremely hot and cold days [17]. All specifications were adjusted for both county and year-month fixed effects. Power plant retirement is estimated to have strong effects on PM_2.5_ concentrations in their downwind counties: it reduced monthly PM_2.5_ concentrations by 2.1 *μg*/*m*^3^. The estimated coefficients of the IV are stable across all three specifications.

Columns (4)–(6) present the main results of PM_2.5_ on mortality using power plant retirement as an instrument variable for PM_2.5_ concentrations. The control variables and county and year-month fixed effects are the same as in the first stage. PM_2.5_ concentrations are estimated to have strong effects on mortality and are robust to weather and socioeconomic controls. We found that the reduction of PM_2.5_ significantly decreases monthly age-adjusted mortality by 7.17 people per 100,000 or 1.7% (Column (6) of Table 2).

### Heterogeneity and robustness checks

Table 4 presents the gender-specific estimates. The impact of PM_2.5_ concentrations on the male mortality rate is greater than that for females, with the estimated effects of a 1 *μg*/*m*^3^ reduction in monthly PM_2.5_ concentrations leading to 9.9 and 5.5 fewer deaths per 100,000 among males and females respectively.

**Table 4:** The Effect of PM_2.5_ on Male and Female Mortality Rates

	Male Mortality			Female Mortality		
	(1)	(2)	(3)	(4)	(5)	(6)
PM_2.5_ (μg/m^3^)	9.30***	9.17***	9.89***	5.00**	4.80**	5.50**
	(4.75–13.85)	(4.60–13.73)	(5.15–14.63)	(0.76–9.25)	(0.63–8.97)	(1.16–9.84)
Weather Controls	N	Y	Y	N	Y	Y
Socioeconomic Controls	N	N	Y	N	N	Y
County Fixed Effects	Y	Y	Y	Y	Y	Y
Month Fixed Effects	Y	Y	Y	Y	Y	Y
Observations	8274	8274	8274	8274	8274	8274

Notes: This table reports the instrumental variable regression coefficients and standard errors. Retirement of coal-fired power plants is used as the instrumental variables for monthly PM2.5 concentrations. The dependent variable is the monthly standardized mortality rate per 100, 000 people. Columns 1–3 and 4–6 show estimates for males and females separately. Weather controls include temperature, dew point, and barometric pressure. Socioeconomic controls include median household income and poverty rate. Standard errors are clustered at the state level. * *p* < 0.10, ** *p* < 0.05, *** *p* < 0.01

We investigated the nonlinear effects across age groups by examining the impact of PM_2.5_ on mortality rates separately for different age groups. The results are reported in Table 5. We estimated the model for the full sample, and separately for males and females. We found that PM_2.5_ concentrations have a large effect on both males and females older than 75 years. For people older than 75, the results show that the impact of a 1- *μg*/*m*^3^ reduction in PM_2.5_ concentrations is 21 and 12 fewer deaths per 100,000 for males and females, respectively. For those ages 65–75, we did not find a statistically significant impact.

**Table 5:** The Effect of PM_2.5_ on Age-Specific Mortalities

	Models		
	(1)	(2)	(3)
	Full Sample		
Age 65–75	−0.53 (−2.41–1.36)	−0.53 (− 2.34–1.27)	−0.04 (− 2.10–2.01)
Age 75+	14.75*** (8.40–21.10)	14.37*** (8.08–20.67)	15.40*** (9.00–21.79)
	Male		
Age 65–75	1.18 (− 4.61–6.97)	1.24 (−4.50–6.97)	1.80 (−4.31–7.92)
Age 75+	20.22*** (12.68–27.75)	19.78*** (12.34–27.23)	20.75*** (13.46–28.03)
	Female		
Age 65–75	−1.55 (− 4.36–1.27)	−1.62 (− 4.48–1.24)	−1.23 (− 3.86–1.40)
Age 75+	11.61** (2.57–20.66)	11.27** (2.29–20.25)	12.32*** (3.20–21.44)
Weather Controls	N	Y	Y
Socioeconomic Controls	N	N	Y
County Fixed Effects	Y	Y	Y
Month Fixed Effects	Y	Y	Y

Notes: This table reports the instrumental variable regression coefficients and standard errors. Each cell represents a separate regression of monthly age-specific mortality rates (deaths per 100,000 people) on PM_2.5_ concentrations (*μg*/*m*^3^). Retirement of coal-fired power plants is used as the instrumental variables for monthly PM_2.5_ concentrations. The dependent variable is the monthly standardized mortality rate per 100, 000 people. The specification corresponds to the column 6 specification in Table 2. Weather controls include temperature, dew point, and barometric pressure. Socioeconomic controls include median household income and poverty rate. Standard errors are clustered at the state level. * *p* < 0.10, ** *p* < 0.05, *** *p* < 0.01

The results for robustness checks are presented in the Appendix. First, we control for county-level annual smoking rates. Appendix Table 7 presents the results which are very similar in both magnitude and statistical significance to the main results. Second, we included fewer matched control counties for each treated county. The estimates in Appendix Table 8 are similar to our main results. Third, we include those power plants that installed a scrubber, as shown in Appendix Table 9. The results are generally consistent with those in the main specifications in Tables 3, 4, 5. Finally, when we excluded the counties that have less than 60 months of data, the results (Appendix Table 10) are similar to our main results.

### Direct impact of power plant retirement on air pollution and mortality

We also directly estimated the impact of coal power plant retirement on mortality using the DID strategy. Table 6 reports the results. The results show that the impact of coal power plant retirement decreased mortality by 15 per 100,000 or 3.6%, as well as decreases PM_2.5_ concentration by 2.13 μg/m^3^. The effect was higher for males than females and is mainly driven by fewer deaths among people older 75 years. These results are consistent with what we found using the IV approach.

**Table 6:** The Effect of Power Plant Shutdown on PM2.5 and Mortalities

Dependent Variables	Models		
	(1)	(2)	(3)
	Effect of Plant Shutdown on Air Pollution		
PM_2.5_ (μg/m3)	−2.11*** (− 2.78 - -1.44)	−2.13*** (− 2.82 - -1.44)	−2.13*** (− 2.84 - -1.43)
	Effect of Plant Shutdown on Mortality		
Overall Mortality	−13.97*** (− 21.04 - -6.89)	− 13.74*** (− 20.27 - -7.22)	−15.31*** (− 21.80 - -8.82)
Male Mortality	− 19.63*** (− 30.30 - -8.96)	− 19.53*** (− 29.75 - -9.32)	−21.10*** (− 31.61 - -10.59)
Female Mortality	− 10.56** (− 19.07 - -2.04)	−10.22** (− 18.48 - -1.97)	−11.74*** (− 20.03 - -3.45)
Mortality, 65–75	1.11 (− 3.26–5.49)	1.14 (− 3.15–5.42)	0.09 (− 4.68–4.86)
Mortality, 75+	−31.13*** (− 42.98 - -19.28)	−30.63*** (− 41.56 - -19.71)	−32.85*** (− 43.17 - -22.54)
Weather Controls	N	Y	Y
Socioeconomic Controls	N	N	Y
County Fixed Effects	Y	Y	Y
Month Fixed Effects	Y	Y	Y

Notes: This table reports the difference-in-differences regression coefficients and standard errors. The dependent variable is PM_2.5_ concentrations (*μg*/*m*^3^) in the row and the monthly standardized mortality rate per 100, 000 people in other rows. Weather controls include temperature, dew point, and barometric pressure. Socioeconomic controls include median household income and poverty rate. Standard errors are clustered at the state level. * *p* < 0.10, ** *p* < 0.05, *** *p* < 0.01

## Discussion

Using the IV approach, we found that the reduction of PM_2.5_ significantly decreased monthly age-adjusted mortality by 7 per 100,000 or 1.7% of the mortality rate. Using the DID approach, we found that the retirement of power plants significantly decreased monthly age-adjusted mortality by 15 per 100,000 or 3.6% of the mortality rate. The effect on mortality was greater for males than for females. When we examined different age groups, we found the results were driven by reduced mortality among people older than 75 years.

A critical question of PM research is to identify the magnitude of public health benefits from reduction of particulates [18]. Here, we assessed the benefit of lowering PM_2.5_ concentrations in the US. According to the 2010 Census, there are 38,613,000 people older than 65 in the US.^1^ Using the most comprehensive IV estimate in Column (6) of Table 2, which reports that the reduction of PM_2.5_ significantly decreases monthly age-adjusted mortality by 7.17 people over 100,000, a back-of-envelope calculation shows that 3322 (95% CI 1622, 5027) deaths per year could have been avoided among the US population older than 65 if PM_2.5_ concentrations decreased by 1 *μg*/*m*^3^.

The general magnitude of our results aligns with previous studies [19, 20], but there is inconsistency among results that have been reported thus far between various studies and even within the same study using different causal methods. Using IV analysis, Schwartz et al. found a 1.54% increase in daily deaths per 10 μg/m^3^ PM_2.5_ increase. In contrast, they reported that the marginal structural model estimate for PM_2.5_ was 0.75% for the same increment [19]. In another study by Schwartz et al. 2015, a 0.5% daily mortality increase was reported to be a result of a 1 μg/m^3^ PM_2.5_ increase using IV and propensity score methods [20]. These effects are found at a low level of PM_2.5_ both above and below the current EPA standard (12 μg/m^3^). Other natural experiment studies of short-term PM_2.5_ exposure focused on birth weight, reporting a significant reduction in birth weight due to short-term exposure to PM_2.5_ in Beijing and in communities near a New Jersey power plant [21, 22].

Here, we have used both the IV and DID approaches. Our results are consistent across the two approaches. Our study has several limitations. First, we did not have detailed information on pollutant emissions at each power plant, which could have further validated our assumption that each power plant underwent retirement at the year and month we identified. Lacking access to emission data could potentially bias our DID estimates if emission reduction proceeds the official retirement of the coal-fired power plants. In such case, we will end up under-estimating the effect of the retirement of the coal-fired power plants. Second, having no data on disease-specific mortality, such as cardiovascular and/or respiratory disease, reduced the sensitivity of our outcome measures to air pollution. Third, we did not have access to mortality data after 2013, restricting the size of our treatment group. However, the fact that we were able to identify an effect increases the validity of our natural experiment. Fourth, details of exact scrubber installation are not always available for each power plant and can be ambiguous even when they are available; lack of accurate air pollution control measures at the plant level reduced the power of our analysis. Fifth and finally, we conducted robustness checks to show that our instrument is valid: retirement of coal-fired plants affects mortality through air pollution only. However, we are not able to completely rule out other factors that might affect mortality.

## Conclusions

We applied IV approach in a natural experiment setting at the individual power plant level to estimate the causal effect of PM_2.5_ concentrations on mortality rates among U.S. adults older than 65 years. We used DID method to directly estimate the effects of power plant retirements on PM_2.5_ concentrations and monthly mortality among U.S. adults older than 65 years. We conclude that power plant retirements lead to a significant reduction in PM_2.5_ concentrations and consequently decreases monthly mortality rates among U.S. adults older than 65. The mortality effects are higher among males than females and are driven by fewer deaths among people who are over 75 years old.

## Figures and Tables

**Fig. 3:**
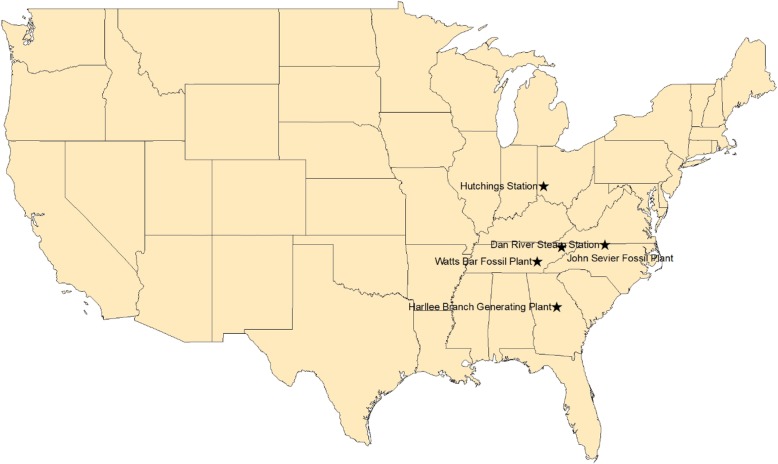
Map of Power Plants

## Data Availability

CMS data we used in the paper are confidential. Pollution, weather, and socio-economic data are available.

## Appendices

**Table 7:** The Effect of PM_2.5_ on Mortalities (Control for County-level Annual Smoking Rates)

	Models		
	(1)	(2)	(3)
	Full Sample		
PM_2.5_ (μg/m3)	6.54*** (3.06–10.02)	6.37*** (2.94–9.80)	6.91*** (3.20–10.63)
	Male		
PM_2.5_ (μg/m3)	8.99*** (4.45–13.52)	8.91*** (4.34–13.48)	9.49*** (4.59–14.39)
	Female		
PM_2.5_ (μg/m3)	5.09** (0.96–9.22)	4.84** (0.78–8.91)	5.34** (1.06–9.63)
	Age Groups		
PM_2.5_ (μg/m3)			
Age 65–75	−0.29 (− 2.11–1.53)	−0.29 (− 2.04–1.47)	0.07 (− 1.97–2.11)
Age 75+	14.35*** (8.24–20.46)	13.97*** (7.90–20.03)	14.74*** (8.34–21.14)
Weather Controls	N	Y	Y
Socioeconomic Controls	N	N	Y
County Fixed Effects	Y	Y	Y
Month Fixed Effects	Y	Y	Y

Notes: This table reports the instrumental variable regression coefficients and standard errors. Retirement of coal-fired power plants is used as the instrumental variables for monthly PM_2.5_ concentrations. The dependent variable is the monthly standardized mortality rate per 100, 000 people. We control for county-level annual smoking rates in this robustness check. Weather controls include temperature, dew point, and barometric pressure. Socioeconomic controls include median household income and poverty rate. Standard errors are clustered at the state level. * *p* < 0.10, ** *p* < 0.05, *** *p* < 0.01

**Table 8:** The Effect of PM_2.5_ on Mortalities (Different Number of Matched Control Counties)

	Models		
	(1)	(2)	(3)
	Full Sample		
PM_2.5_ (μg/m3)	7.85*** (4.02–11.68)	7.68*** (3.80–11.56)	8.16*** (3.87–12.45)
	Male		
PM_2.5_ (μg/m3)	11.50*** (6.22–16.77)	11.27*** (5.81–16.72)	11.50*** (5.82–17.19)
	Female		
PM_2.5_ (μg/m3)	5.50*** (1.40–9.59)	5.35** (1.25–9.44)	5.99** (1.22–10.76)
	Age Groups		
PM_2.5_ (μg/m3)			
Age 65–75	− 0.85 (−3.40–1.71)	−0.85 (− 3.40–1.71)	−0.84 (− 3.58–1.91)
Age 75+	17.65*** (11.20–24.11)	17.25*** (10.67–23.82)	18.29*** (11.01–25.58)
Weather Controls	N	Y	Y
Socioeconomic Controls	N	N	Y
County Fixed Effects	Y	Y	Y
Month Fixed Effects	Y	Y	Y

Notes: This table reports the instrumental variable regression coefficients and standard errors. Retirement of coal-fired power plants is used as the instrumental variables for monthly PM_2.5_ concentrations. The dependent variable is the monthly standardized mortality rate per 100, 000 people. We matched only 5 (vs. 10 in main analyses) control counties with each treatment county in this robustness check. Weather controls include temperature, dew point, and barometric pressure. Socioeconomic controls include median household income and poverty rate. Standard errors are clustered at the state level. * *p* < 0.10, ** *p* < 0.05, *** *p* < 0.01

**Table 9:** The Effect of PM_2.5_ on Mortalities (Include Power Plants with Scrubbers)

	Models		
	(1)	(2)	(3)
	Full Sample		
PM_2.5_ (μg/m3)	8.47** (1.62–15.31)	8.22** (1.50–14.94)	8.85*** (2.33–15.38)
	Male		
PM_2.5_ (μg/m3)	11.29*** (4.83–17.75)	11.11*** (4.67–17.56)	11.72*** (5.38–18.06)
	Female		
PM_2.5_ (μg/m3)	6.71* (−0.81–14.23)	6.39* (− 0.94–13.72)	7.02* (− 0.10–14.13)
	Age Groups		
PM_2.5_ (μg/m3)			
Age 65–75	−0.54 (− 2.48–1.41)	−0.53 (− 2.39–1.34)	−0.09 (− 2.11–1.93)
Age 75+	18.75*** (5.27–32.24)	18.19*** (4.88–31.49)	19.08*** (6.26–31.90)
Weather Controls	N	Y	Y
Socioeconomic Controls	N	N	Y
County Fixed Effects	Y	Y	Y
Month Fixed Effects	Y	Y	Y

Notes: This table reports the instrumental variable regression coefficients and standard errors. Retirement of coal-fired power plants is used as the instrumental variables for monthly PM_2.5_ concentrations. The dependent variable is the monthly standardized mortality rate per 100, 000 people. We include power plants with installed scrubber. Weather controls include temperature, dew point, and barometric pressure. Socioeconomic controls include median household income and poverty rate. Standard errors are clustered at the state level. * *p* < 0.10, ** *p* < 0.05, *** *p* < 0.01

**Table 10:** The Effect of Power Plant Shutdown on PM_2.5_ and Mortalities (Excluding Counties with Less Than 60 Months of Data)

	Models		
	(1)	(2)	(3)
	Full Sample		
PM_2.5_ (μg/m3)	6.62*** (3.05–10.19)	6.45*** (2.94–9.96)	7.14*** (3.45–10.83)
	Male		
PM_2.5_ (μg/m3)	9.29*** (4.74–13.84)	9.15*** (4.60–13.69)	9.81*** (5.08–14.55)
	Female		
PM_2.5_ (μg/m3)	5.00** (0.76–9.25)	4.82** (0.64–8.99)	5.50** (1.14–9.86)
	Age Groups		
PM_2.5_ (μg/m3)			
Age 65–75	−0.52 (− 2.40–1.36)	− 0.52 (− 2.32–1.27)	− 0.02 (− 2.06–2.03)
Age 75+	14.74*** (8.39–21.08)	14.37*** (8.08–20.66)	15.30*** (8.85–21.74)
Weather Controls	N	Y	Y
Socioeconomic Controls	N	N	Y
County Fixed Effects	Y	Y	Y
Month Fixed Effects	Y	Y	Y

Notes: This table reports the instrumental variable regression coefficients and standard errors. Retirement of coal-fired power plants is used as the instrumental variables for monthly PM_2.5_ concentrations. The dependent variable is the monthly standardized mortality rate per 100, 000 people. We excluded those counties with less than five years of data. Weather controls include temperature, dew point, and barometric pressure. Socioeconomic controls include median household income and poverty rate. Standard errors are clustered at the state level. * *p* < 0.10, ** *p* < 0.05, *** *p* < 0.01

## Footnotes

1 The population size data are access from the Census website: https://factfinder.census.gov/faces/tableservices/jsf/pages/productview.xhtml?src=CF.
